# Sustained elevation of NF-κB activity sensitizes offspring of maternal inflammation to hypertension *via* impairing PGC-1α recovery

**DOI:** 10.1038/srep32642

**Published:** 2016-09-12

**Authors:** Yafei Deng, Qi Zhang, Hongqin Luo, Xianhua Chen, Qi Han, Fangjie Wang, Pei Huang, Wenjing Lai, Xiao Guan, Xiaodong Pan, Yan Ji, Wei Guo, Ling Che, Yuan Tang, Liangqi Gu, Jianhua Yu, Michael Namaka, Youcai Deng, Xiaohui Li

**Affiliations:** 1 Institute of Materia Medica, College of Pharmacy, Third Military Medical University, Chongqing, China; 2 Center of Translational Medicine, College of Pharmacy, Third Military Medical University, Chongqing, China; 3 Diagosis and Treatment Center for Servicemen, Southwest Hospital, Third Military Medical University, Chongqing, China; 4 The Center for Disease Control and Prevention of Chengdu Military Command, Chengdu, China; 5 Division of Hematology, Department of Internal Medicine, The Ohio State University, Columbus, Ohio, USA; 6 Colleges of Pharmacy and Medicine, University of Manitoba, Winnipeg, MB, Canada; 7 Joint Laboratory of Biological Psychiatry Between Shantou University Medical College and the College of Medicine University of Manitoba, Shantou, China

## Abstract

Growing evidence has demonstrated that maternal detrimental factors, including inflammation, contribute to the development of hypertension in the offspring. The current study found that offspring subjected to prenatal exposure of inflammation by lipopolysaccharide (LPS) challenge during the second semester showed significantly increased systolic blood pressure. In addition, these offspring also displayed augmented vascular damage and reactive oxygen species (ROS) levels in thoracic aortas when challenged with deoxycorticosterone acetate and high-salt diet (DOCA-salt). Interestingly, the antioxidant N-acetyl-L-cysteine markedly reversed these changes. Mechanistically, prenatal LPS exposure led to pre-existing elevated peroxisome proliferators-activated receptor-γ co-activator (PGC)-1α, a critical master of ROS metabolism, which up-regulated the ROS defense capacity and maintained the balance of ROS generation and elimination under resting state. However, continued elevation of NF-κB activity significantly suppressed the rapid recovery of PGC-1α expression response to DOCA-salt challenge in offspring that underwent prenatal inflammatory stimulation. This was further confirmed by using a NF-κB inhibitor (N-p-Tosyl-L-phenylalanine chloromethyl ketone) that restored PGC-1α recovery and prevented blood pressure elevation induced by DOCA-salt. Our results suggest that maternal inflammation programmed proneness to NF-κB over-activation which impaired PGC-1α-mediated anti-oxidant capacity resulting in the increased sensitivity of offspring to hypertensive damage.

Cardiovascular disease (CVD) represents the ultimate source of morbidity, mortality and healthcare cost worldwide. Hypertension, the leading remediable risk factor for CVD, causes 9.4 million deaths annually worldwide^1^. Unfortunately, the percentage of hypertensive patients with adequate blood pressure control is only about 13%^2^. Therefore, the attempt to uncover the causality and pathogenesis of hypertension may provide new biological targets for which novel therapeutic agents can be developed to control and prevent hypertension.

Compared to the concept of the conventional cardiovascular risk factors in adult life, early life exposure to detrimental factors, such as inflammatory stimuli, obesity, smoking, low-protein diet and abuse of alcohol, has been implicated in the development of CVD in adults^3^. Prenatal exposure to inflammation is still an important unresolved public health problem worldwide, including bacterial or virus infection, chorioamnionitis, periodontitis, rheumatoid arthritis and others^4^,^5^,^6^. We previously established a solid prenatally programmed hypertension (PPH) animal model by stimulating pregnant Sprague-Dawley (SD) rats with lipopolysaccharide (LPS) or zymosan during the secondary semester, a critical period for fetal cardiovascular system development^7^,^8^,^9^. Although we have found intra-renal renin angiotensin system over-activation as well as the presence of vascular remodeling in adult offspring of LPS-treated mothers prior to the onset of hypertension^7^,^10^, the molecular mechanisms are still largely unknown.

Recent studies have also showed that exposure to several detrimental factors during pregnancy, such as protein restriction or diabetes, has been reported to sensitize offspring to the development of hypertension when subjected to conventional hypertensive risk factors in adulthood^11^,^12^. However, whether and how prenatal inflammation stimulation results in increased susceptibility of hypertension responding to hypertensive risk factors are still a question. What’s more, advanced understanding of the mechanisms of prenatal detrimental factors exposure sensitizing adult offspring to hypertension may provide an alternative strategy to treat and prevent hypertension.

Our recent findings showed that early life nuclear factor-κB (NF-κB) dyshomeostasis^13^ and also an increased reactive oxygen species (ROS)^14^ existed in vasculature or heart of offspring of LPS-treated mothers, respectively. Peroxisome proliferators-activated receptor-γ co-activator -1α (PGC-1α) is a broad and powerful regulator of ROS metabolism through co-regulating the induction of proteins that participate in the cellular response to oxidative stress^15^. Also, several studies have established the crosstalk between NF-κB and PGC-1α in management of inflammation and oxidative stress^16^,^17^, which prompted us to explore whether this signaling crosstalk participates in the susceptibility of hypertension responding to hypertensive risk factors in our model.

To further explore the molecular signaling pathway involved in offspring’s hypertension (induced by prenatal exposure to inflammation), we challenged adult offspring of LPS-treated mothers with an aldosterone analogue, deoxycorticosterone acetate (DOCA) in order to determine the susceptibility of hypertension and its related mechanisms. Interestingly, primary hyper-aldosteronism accounts for 5 to 18% of adult hypertension and 14 to 21% of resistant hypertension^18^,^19^. Here we show that prenatal inflammatory stimulus predisposed offspring to hypertensive challenge through DOCA-salt treatment. Mechanistically, continued elevation of NF-κB activity impaired PGC-1α recovery, resulting in reduced anti-oxidative capacity in adult offspring of LPS-treated mothers. This in turn contributed to vascular damage thereby implicating this molecular pathway in the development of hypertension. As such, these findings propose an alternative mechanism involved in prenatal inflammatory stimulus-induced hypertension and provide novel interventional opportunities to control the development of hypertension.

## Results

### Maternal inflammation aggravates vascular damage and hypertension in adult offspring after DOCA-salt treatment

**Figure 1:**
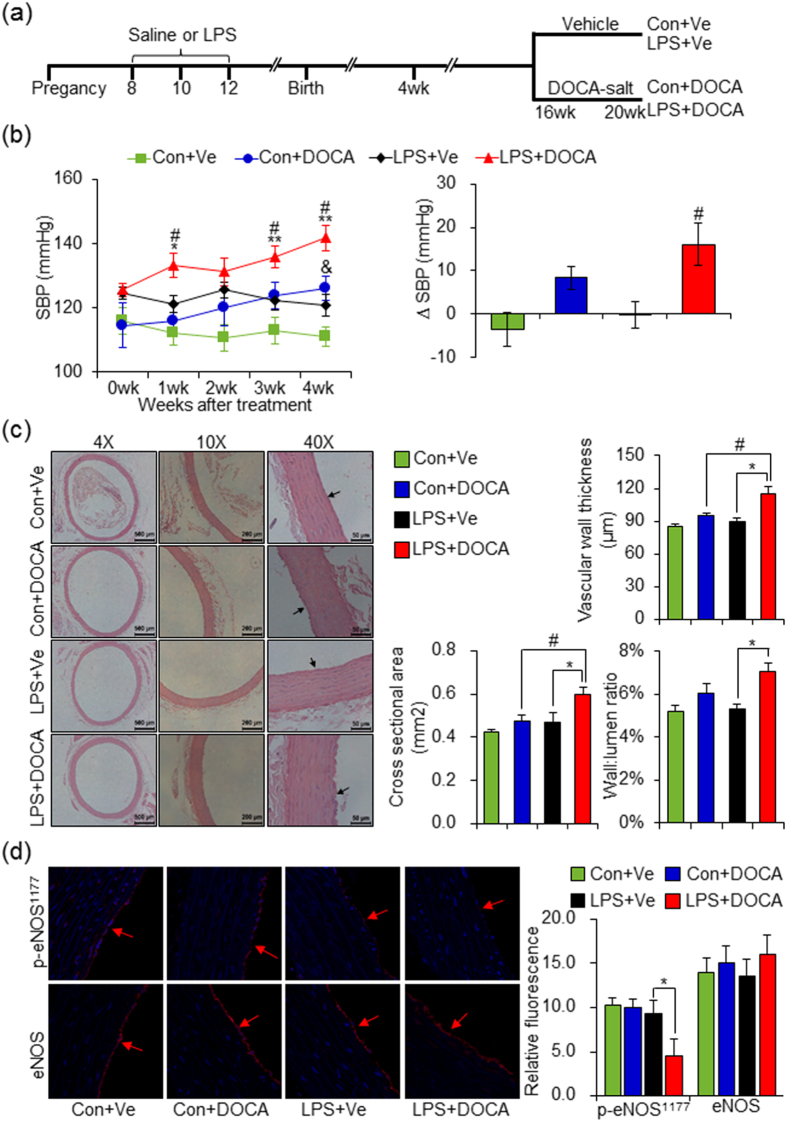
Augmented elevation of systolic blood pressure (SBP) and vascular damage in adult offspring of LPS-treated mothers after 4 weeks of DOCA-salt treatment. (**a**) Schematic diagram of experimental design. Pregnant SD rats were administered intraperitoneally (i.p) with saline or lipopolysaccharide (LPS, 0.79 mg/kg) at gestational day (GD) 8, 10 and 12. Offspring were challenged with deoxycorticosterone acetate and high-salt diet (DOCA-salt) every other day (5–6 pm) for 4 weeks that started at the age of 16 weeks. (**b**) SBP was measured by a noninvasive tail-cuff method at 9–11 am the next day after DOCA treatment at indicated time points after DOCA-salt treatment (left panel). 0 wk means the day before the first time DOCA treatment. The increment of SBP at the end of 4 weeks DOCA-salt treatment in each group (∆SBP) was shown in the right panel. n = 10 to 12 per group. (**c**) Hematoxylin-eosin (HE) staining of thoracic aortas and representative pictures from each group were shown. Vessel wall, nearby the arrow direction, represents endothelium. The values of vascular wall thickness, cross sectional area and wall:lumen ratio were quantified using NIS-Elements BR software (Nikon Corporation, Tokyo, Japan) and shown in the right panel. n = 6 per group. (**d**) Offspring were treated as described in (**a**) and the p-eNOS^Ser1177^ and eNOS protein levels were determined by immunofluorescence staining. Representative pictures selected from each group were shown. Vessel wall, nearby the arrow direction, represents endothelium. The relative fluorescence in each group was quantified using Image J software (right panel). n = 6 per group. Error bar represents S.E.M. *P < 0.05 or **P < 0.01, LPS+DOCA vs LPS+Ve at the same time point, respectively; ^#^*P* < 0.05, LPS+DOCA vs Con+DOCA at the same time point; ^&^*P* < 0.05, Con+DOCA vs Con+Ve at the same time point. Two-way ANOVA.

Recent findings have demonstrated that prenatal exposure to detrimental factors sensitizes offspring to hypertension when challenging with high-salt diet^12^ or angiotensin II^20^. We first exploited the use of DOCA, an aldosterone analogue that induces the animals to a state of hypertension when combined with unilateral nephrectomy in rats^21^, as a “second hit” to evaluate the hypertensive sensitivity in adult offspring of LPS-treated mothers. At the age of 16 weeks, both control and offspring of prenatal LPS exposure received 1% NaCl in drinking water together with subcutaneous injection of DOCA every other day for 4 weeks, defined as Con+DOCA and LPS+DOCA group, respectively. Control and offspring of prenatal LPS exposure administrated with 50% glycerol subcutaneously alone were taken as vehicle control, defined as Con+Ve and LPS+Ve group, respectively (Fig. 1a). DOCA-salt treated adult offspring of LPS-treated mothers showed a significantly elevated systolic blood pressure (SBP) after 1 week of DOCA-salt treatment (LPS+DOCA vs LPS+Ve, *P* < 0.05) and the SBP continued to rise during the whole period of DOCA-salt treatment. The control offspring treated with DOCA-salt, however, just showed a slight increase of SBP after the first week of DOCA-salt treatment and showed statistical significance at the end of DOCA-salt treatment, as compared to that in control offspring with vehicle treatment (Con+DOCA vs Con+Ve, *P* < 0.05) (Fig. 1b, left panel). Remarkably, at the end of 4 weeks of DOCA-salt treatment, the increment of SBP in adult offspring of LPS-treated mothers was greater than that in control offspring (Fig. 1b, right panel). These results suggested a greater hypertensive response in offspring of prenatal exposure to inflammatory stimulus when challenged with second hypertensive risk factors.

We next determined the vascular structures of both thoracic aortas and superior mesenteric arteries by hematoxylin-eosin (HE) staining at the end of 4 weeks of DOCA-salt treatment. Data showed that compared with vehicle control, obvious increases in arterial vascular wall thickness, cross sectional area, wall:lumen ratio and asperous endothelial layers existed in thoracic aortas of adult offspring of LPS-treated mothers after DOCA-salt treatment (LPS+DOCA vs LPS+Ve, *P* < 0.05). Interestingly, we also noticed some slight changes in control offspring after DOCA treatment (Fig. 1c). In addition, the arterial wall thickness and cross sectional area significantly increased in adult offspring of LPS-treated mothers after DOCA treatment, as compared to these in DOCA-salt treated control offspring (LPS+DOCA vs Con+DOCA, *P* < 0.05). We also found similar changes in superior mesenteric arteries after DOCA-salt treatment ((Supplementary Fig. S1). Endothelium dysfunction, classically characterized by a decrease in endothelial nitric oxide synthase (eNOS) bioactivity and a lack of nitric oxide (NO)^22^, is associated with hypertension. As such, the expression of phosphorylated (p)-eNOS^Ser1177^ protein in thoracic aortas was determined by immunofluorescence staining at the end of 4 weeks of DOCA-salt treatment. The p-eNOS^Ser1177^ protein level in control offspring was not changed after DOCA-salt treatment, but decreased after DOCA-salt treatment in adult offspring of LPS-treated mothers (Fig. 1d). These results demonstrated that maternal inflammation results in higher vulnerability to vascular damage and hypertension in adult when suffered from hypertensive risk factors.

### Oxidative stress enhanced hypertensive response to DOCA-salt treatment in offspring of maternal inflammation *via* impairing the up-regulation of antioxidant capacity

**Figure 2:**
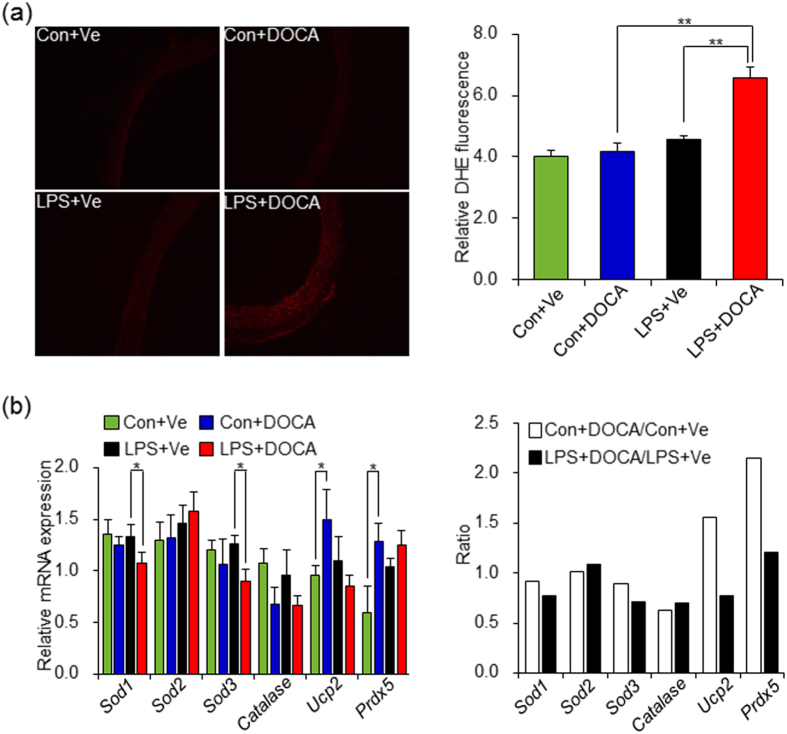
Oxidative stress mediated by impaired up-regulation of antioxidant capacity exists in the thoracic aortas of adult offspring of LPS-treated mothers after 4 weeks of DOCA-salt treatment. (**a**) *In situ* O^2−^ level in thoracic aortas was detected by dihydroethidium (DHE) staining. Representative pictures from each group were shown (left panel) and the value of DHE fluorescence was quantified using Image J software (right panel). Error bar represents S.E.M. **P < 0.01 denotes the statistical comparison between the two marked treatment groups. n = 5 per group. (**b**) The mRNA levels of antioxidant-related genes in thoracic aortas were assessed by real-time reverse transcription PCR (RT-PCR). *β-actin* was taken as internal control. Error bar represents S.E.M. *P < 0.05 denotes the statistical comparison between the two marked treatment groups, respectively. n = 6–7 per group. Two-way ANOVA.

Experimental models and circulating markers screening of systemic oxidative stress in human have suggested that oxidative stress promotes endothelial dysfunction, vascular remodeling, which correlates positively with SBP elevation^23^. Previous studies have shown that oxidative stress was implicated in traditional DOCA-salt induced hypertension model that with uninephrectomy^24^ and also in adult offspring that had maternal exposure to nicotine and high-fat diet^25^,^26^. This raises the possibility that prenatal exposure to LPS impairs offspring’s redox homeostasis and predisposes to DOCA-salt challenge. As such, we first determined the *in situ* O^2−^ level by dihydroethidium (DHE) staining of thoracic aortas. The fluorescence intensity was similar between vehicle-treated control and adult offspring of LPS-treated mothers and was not increased in control offspring after DOCA-salt treatment. However, the level of O^2−^ in adult offspring of LPS-treated mothers was higher than that in control offspring after 4 weeks of DOCA-salt treatment (LPS+DOCA vs Con+DOCA, *P* < 0.01) (Fig. 2a). As such, this finding supported the hypothesis that exaggerated oxidative stress exists in adult offspring of LPS-treated mothers after DOCA-salt treatment.

We further explored the potential mechanisms of exaggerated oxidative stress in thoracic aortas of offspring that received prenatal exposure to LPS after DOCA-salt treatment from two aspects: reactive oxygen species (ROS) generation and cellular antioxidant defense system, whose imbalance contributes to vascular oxidative stress^27^. Firstly, we measured the mRNA levels of NADPH oxidase subunits, the major enzyme of ROS generation in the artery wall, many of which are involved in hypertension^28^. The mRNA expression of these genes did not significant change, although some of them, such as *Nox1, p22-phox, p47phox* and *p67phox* (*Ncf2*) showed an increased trend in either control offspring or adult offspring of LPS-treated mothers after DOCA-salt treatment (Supplementary Fig. S2a). These findings prompted us to investigate whether the anti-ROS capacity changes or not after DOCA-salt treatment by determining the expression of superoxide dismutases (SODs), heme oxygenase (HMOX-1), catalase, glutathione reductase-1(GPx-1), peroxiredoxins (Prdxs), and uncoupling protein 2 (UCP2), all of which have been involved in development of hypertension^29^. There were no significant changes of *Gpx1, Hmox1*, as well as *Prdx3* mRNA expression in offspring of both control and prenatal exposure to LPS after DOCA-salt treatment, respectively (Supplementary Fig. S2b). Surprisingly, the mRNA levels of *Sod1* and *Sod3* significantly decreased in thoracic aortas of adult offspring of LPS-treated mothers after DOCA-salt treatment, as compared to that in vehicle treated alone (LPS+DOCA vs LPS+Ve, *P* < 0.05). However, there were no significant changes of these two genes in thoracic aortas between DOCA-salt and vehicle treated control offspring (Con+DOCA vs Con+Ve, *P* > 0.05). Interestingly, the mRNA levels of *Ucp2* and *Prdx5* significantly increased in thoracic aortas in control offspring after DOCA-salt treatment (Con+DOCA vs Con+Ve, *P* < 0.05) but with no significant changes after DOCA-salt treatment in adult offspring of LPS-treated mothers (LPS+DOCA vs LPS+Ve, *P* > 0.05) (Fig. 2b). These data suggested an impaired ROS defense capacity in response to a second risk factor challenge in offspring of prenatal exposure to inflammatory stimulation.

**Figure 3:**
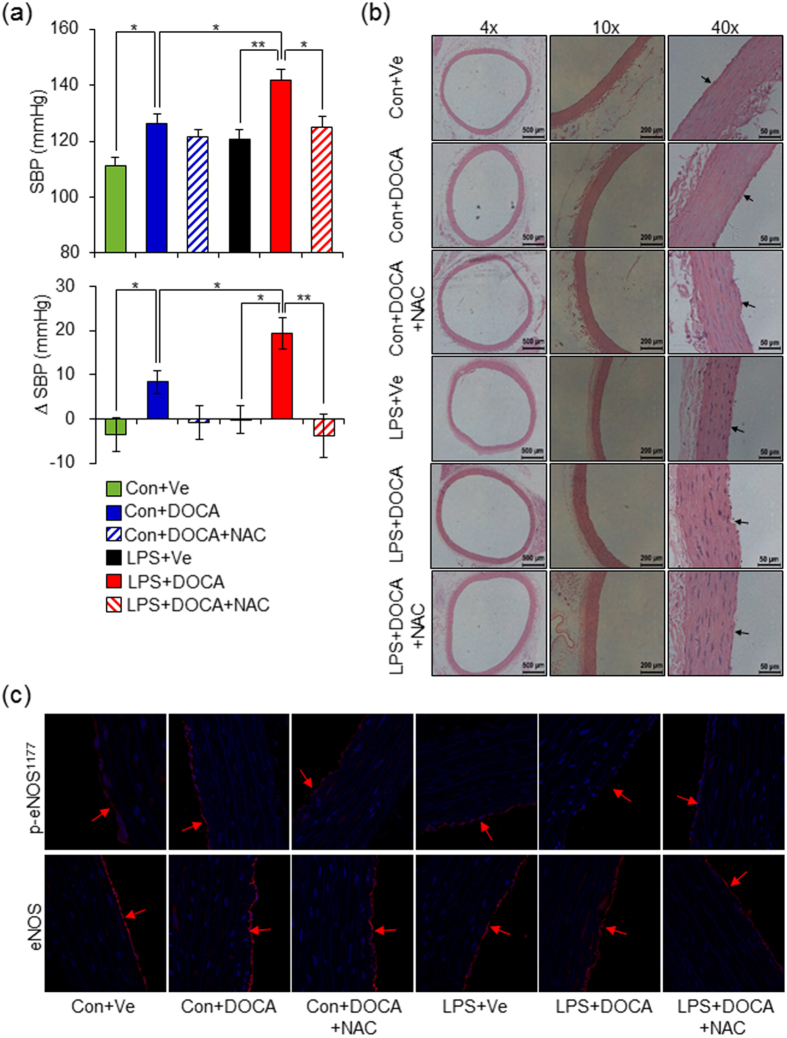
Antioxidant N-acetyl-L-cysteine (NAC) decreases blood pressure and prevents vascular damage in thoracic aortas of offspring that received prenatal exposure to LPS after 4 weeks of DOCA-salt treatment. (**a**) NAC was given simultaneously with DOCA-salt for 4 weeks in both control (Con+DOCA+NAC group) and adult offspring of LPS-treated mothers (LPS+DOCA+NAC group) and SBP was determined by a noninvasive tail-cuff method. (**b**) Offspring were treated as described in (**a**) and HE staining of thoracic aortas and representative pictures from each group were shown. Vessel wall, nearby the arrow direction, represents endothelium. The values of vascular wall thickness, cross sectional area and wall:lumen ratio were quantified using NIS-Elements BR software and were shown in Supplemental Fig. S3a. (**c**) Offspring were treated as described in (**a**) and the p-eNOS^Ser1177^ and eNOS protein levels were determined by immunofluorescence staining. Representative pictures selected from each group were shown. Vessel wall, nearby the arrow direction, represents endothelium. The value of fluorescence was quantified using Image J software and shown in supplemental Fig. S3b. Error bar represents S.E.M. *P < 0.05 and **P < 0.01 denote the statistical comparison between the two marked treatment groups, respectively. n = 6–7 per group for (**a–c**). Two-way ANOVA.

To further confirm the role of oxidative stress in mediating hypertensive response in adult offspring of LPS-treated mothers when challenged with DOCA-salt, a thiol-containing antioxidant, N-Acetyl-L-cysteine (NAC)^30^ was given simultaneously with DOCA-salt treatment. As expected, NAC administration significantly repressed the SBP elevation induced by DOCA-salt treatment in adult offspring of LPS-treated mothers (Fig. 3a). Consistent with these findings, antioxidant NAC prevented DOCA-salt induced increased artery wall thickness, cross sectional area as well as wall:lumen ratio and the damage to endothelial cell layers in adult offspring of LPS-treated mothers (Fig. 3b and Supplemental Fig. S3a) and also rescued the p-eNOS^Ser1177^ protein level (Fig. 3c and Supplementary Fig. S3b). Taken together, these results demonstrated that augmented oxidative stress resulting from the decreased antioxidant capacity is responsible for higher hypertensive response induced by DOCA-salt treatment in offspring of prenatal exposure to inflammatory stimulation and elimination of excessive ROS by NAC could be potent avenue to prevent vascular damage and the development of hypertension in PPH models.

### Impaired PGC-1α recovery by continued elevation of NF-κB activity decreased ROS defense system in adult offspring of LPS-treated mothers in response to DOCA-salt challenge

**Figure 4:**
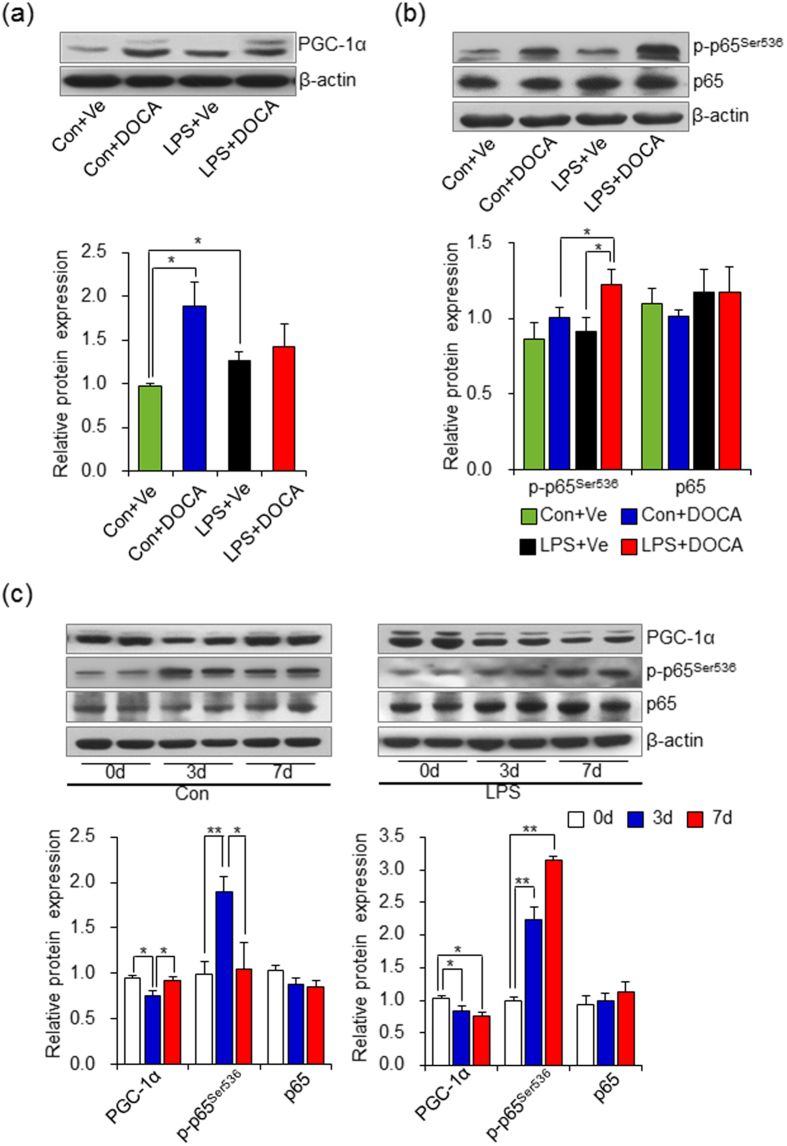
Persistent NF-κB activation retards PGC-1α recovery in thoracic aortas of adult offspring of LPS-treated mothers after DOCA treatment. (**a,b**) Offspring were treated as described in Fig. 1a and the protein expression level of PGC-1α (**a**) and p-p65^Ser536^, p65 (**b**) in thoracic aortas was assessed by immunoblotting after 4 weeks of DOCA-salt treatment. (**c**) Offspring were treated with DOCA-salt by 3 or 7 days and PGC-1α, p-p65^Ser536^ and p65 protein expression in thoracic aortas was assessed by immunoblotting. Representative plots in each group and statistical data of relative densitometry, normalized by β-actin, were shown. n = 6 per group for (**a–c**). Error bar represents S.E.M. *P < 0.05 and **P < 0.01 denotes the statistical comparison between the two marked treatment groups, respectively. Two-way ANOVA.

There are several transcriptional factors that manipulate the balance between ROS and anti-ROS systems^15^. Among these genes, PGC-1α is a broad and powerful regulator of ROS metabolism through co-regulating the induction of proteins that participate in the cellular response to oxidative stress. For example, UCP2 has been proposed to be the direct downstream target of PGC-1α^31^ and Prdx5 expression is mediated by PGC-1α direct association with its promoter regions^32^. Interestingly, knockdown of PGC-1α also significantly decreases SOD1 expression in response to oxidative stress^33^. In our previous data, DOCA-salt treatment led to abnormal expression model of UCP2, prdx5 and SOD1 in adult offspring of LPS-treated mothers (Fig. 2b), suggesting the critical role of PGC-1α in our model. To further address the role of PGC-1α in mediating enhanced oxidative stress in adult offspring of LPS-treated mothers after DOCA-salt treatment, we firstly determined the protein expression of PGC-1α at the end of DOCA-salt treatment. As expected, the protein level of PGC-1α significantly increased after DOCA-salt treatment in thoracic aortas of control offspring (Con+DOCA vs Con+Ve, *P* < 0.05), which was similar with PGC-1α expression response to H_2_O_2_ stimulation challenge as a compensatory mechanism for anti-oxidative stress^33^. However, there was no difference in the expression level of PGC-1α between vehicle and DOCA-salt treated adult offspring of LPS-treated mothers (LPS+DOCA vs LPS+Ve, *P* > 0.05). Besides, the basal level of PGC-1α protein in thoracic aortas of adult offspring of LPS-treated mothers was significantly higher than that in control offspring (LPS+Ve vs Con+Ve, *P* < 0.05) (Fig. 4a). The consistent expression pattern of UCP2, Prdx5 (Fig. 2b) and PGC-1α (Fig. 4a) in our model, strongly suggested that the compensatory action of PGC-1α-mediated enhanced ROS defense system occurs in adult offspring of prenatal exposure to inflammatory stimulation at rest state, but loses its further compensatory capability after a second oxidative stressor challenge. However, it raised an interesting question as to why no PGC-1α increment was observed in offspring of prenatal exposure to inflammatory stimulus after exposure to a hypertensive risk factor?

Several factors affect PGC-1α expression under oxidative stress or inflammatory challenge, such as mitogen activated protein kinase (MAPK) and NF-κB^34^. Among these, MAPK signal pathway has been involved in response to various environmental stresses^35^. Since a previous study found that MAPK signal pathway was activated in traditional DOCA-salt hypertensive rats^36^, we first determined the MAPK signaling in the thoracic aortas of adult offspring of LPS-treated mothers after DOCA-salt treatment. We found that the expression levels of p-p38 MAPK, p-ERK1/2 and p-JNK were not changed in thoracic aortas of both control offspring and adult offspring of LPS-treated mothers after 4 weeks DOCA-salt treatments (Supplementary Fig. S4). Besides, the critical transcriptional factor of inflammatory response, NF-κB, suppresses PGC-1α expression after activated by inflammatory cytokines^37^. Since we previously found increased inflammatory cytokines and NF-κB activation in both kidney and thoracic aortas of adult offspring of LPS-treated mothers^7^,^10^, we assessed the activity of NF-κB by measuring the protein levels of phosphorylated (p)-p65^Ser536^. Interestingly, the p-p65^Ser536^ level peaked in thoracic aortas of adult offspring of LPS-treated mothers after 4 weeks of DOCA-salt treatment, compared to either DOCA-salt treated control offspring or vehicle treated adult offspring of LPS-treated mothers (LPS+DOCA vs Con+DOCA and LPS+DOCA vs LPS+Ve, *P* < 0.05, Fig. 4b). In support of this finding, the expression levels of genes that are downstream targets of NF-κB activity, such as intercellular adhesion molecule-1(ICAM-1), vascular cell adhesion molecule-1(VCAM-1), interleukin-1(IL-1β), interleukin-6 (IL-6) and tumor necrosis factor-alpha (TNF-α), were also increased in thoracic aortas in adult offspring of LPS-treated mothers after 4 weeks of DOCA-salt treatment (Supplementary Fig. S5).

A previous study reported that renal PGC-1α expression was suppressed at the beginning of LPS-induced sepsis and reversed to the normal level during the renal functional recovery and the pattern of PGC-1α expression was believed to be necessary for renal functional recovery after sepsis^38^. This prompted us to determine whether the PGC-1α expression followed the similar pattern at different time-points after DOCA-salt treatment in offspring of both control and adult offspring of LPS-treated mothers and whether NF-κB over-activation was involved in inhibiting PGC-1α expression. In control offspring, we found that the PGC-1α protein expression decreased after 3 days of DOCA-salt treatment but soon rebounded to the initial level by 7 days of DOCA-salt treatment, while p-p65^Ser536^ showed the opposite pattern (Fig. 4c, left panel). However, in adult offspring of LPS-treated mothers, the PGC-1α expression didn’t rebound to the initial level by 7 days of DOCA-salt treatment, meanwhile, p-p65^Ser536^ levels continued to increase from 3 days to 7 days after DOCA-salt treatment (Fig. 4c, right panel). All these data indicated that prenatal exposure to LPS predisposes adult offspring to NF-κB activation and further augmented activation lead to retarded recovery of PGC-1α expression during stressor challenge.

### Inhibition of NF-κB activity by TPCK restores rapid PGC-1α recovery and prevents SBP elevation in adult offspring of LPS-treated mothers in response to DOCA-salt challenge

**Figure 5:**
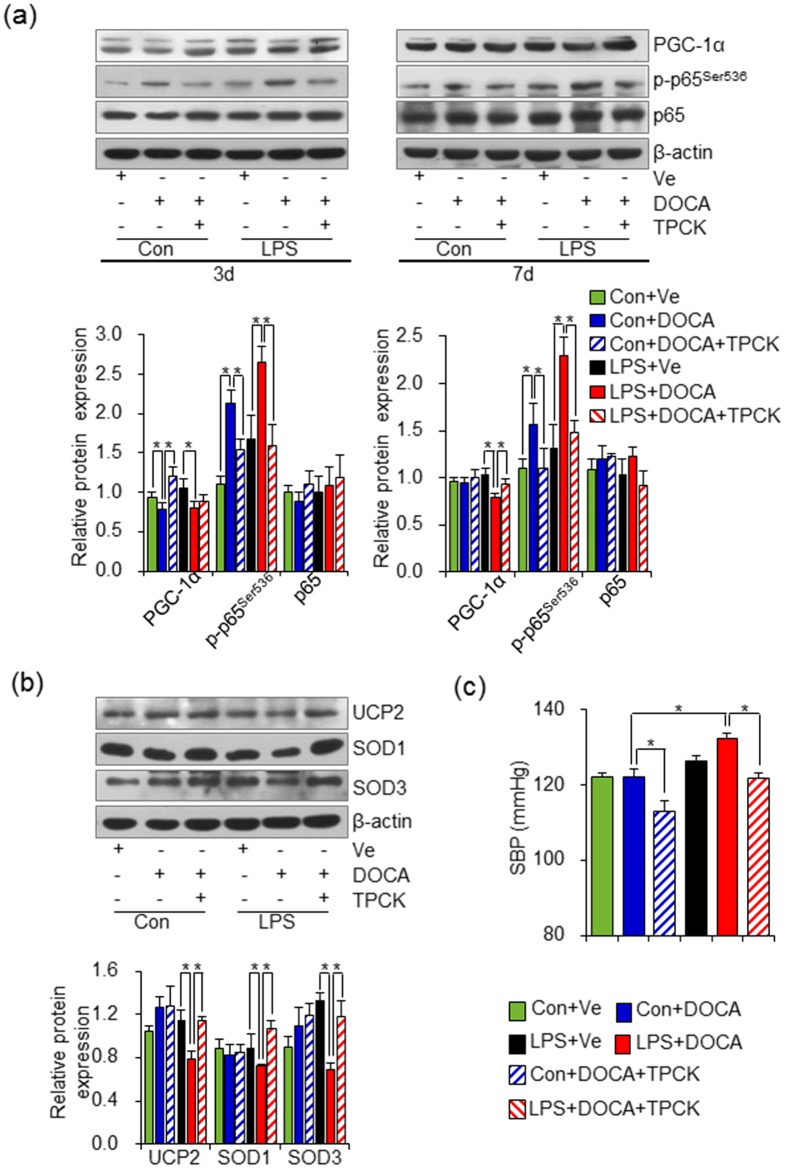
Inhibition of NF-κB over-activation protects PGC-1α rapid recovery and prevents SBP elevation induced by DOCA-salt treatment in adult offspring of LPS-treated mothers. (**a**) NF-κB inhibitor, N-p-Tosyl-L-phenylalanine chloromethyl ketone (TPCK) was daily given simultaneously with DOCA-salt for 3 or 7 days in both control (Con+DOCA+TPCK group) and adult offspring of LPS-treated mothers (LPS+DOCA+TPCK group). Protein expressions of PGC-1α, p-p65^Ser536^ and p65 in thoracic aortas were assessed by immunoblotting. (**b**) Offspring were treated as described in (**a**) for 7 days of TPCK treatment and the protein expression of UCP2, SOD1 and SOD3 in thoracic aortas were assessed by immunoblotting. Representative plots in each group and statistical data of relative densitometry, normalized by β-actin in both (**a,b**) were shown. (**c**) Offspring were treated as described in (**a**) for 7 days of TPCK treatment and SBP were assessed by a noninvasive tail-cuff method. Error bar represents S.E.M. *P < 0.05 and **P < 0.01 denote the statistical comparison between the two marked treatment groups, respectively. n = 6 per group for (**a–c**). Two-way ANOVA.

To further confirm our above hypothesis that the over-activated NF-κB signal pathway in repressing PGC-1α expression in adult offspring of LPS-treated mothers after DOCA-salt treatment, a specific NF-κB inhibitor, N-p-Tosyl-L-phenylalanine chloromethyl ketone (TPCK)^39^, was given simultaneously with DOCA-salt treatment. As expected, TPCK prevented the increased p-p65^Ser536^ level in thoracic aortas of both control offspring and adult offspring of LPS-treated mothers by 3 or 7 days of DOCA-salt treatment. Importantly, TPCK restored PGC-1α expression at 3 days or 7 days after the initial DOCA-salt treatment in thoracic aortas of offspring that underwent prenatal exposure to LPS, respectively (Fig. 5a). TPCK also restored UCP2, SOD1 and SOD3 expression 7 days after the initial DOCA-salt treatment in thoracic aortas of adult offspring of LPS-treated mothers (Fig. 5b). In agreement with the above findings, TPCK administration significantly repressed the SBP increment induced by 7 days of DOCA-salt treatment in adult offspring of LPS-treated mothers (Fig. 5c). All this data demonstrated that prenatal inflammation exposure leads to the proneness of NF-κB activation at resting state and continued elevation of NF-κB activity results in suppressed PGC-1α rebound when facing a stressor challenge, which might be a pivotal factor implicated in vascular damage and progressive development of hypertension *via* an excessive oxidative stress mechanism.

## Discussion

The early onset of CVD is dramatically increasing in the past several years. More than 17% of deaths caused by CVD occurred before the age of 60 in 2012^40^. As a major cardiovascular risk factor, the incidence of raised blood pressure (defined as systolic and/or diastolic blood pressure ≥140/90 mmHg) in adults aged 18 years and over is around 22%^40^. However, hypertension development begins decades before the onset of clinical manifestations and may be hastened by maternal detrimental factors^20^,^41^. Therefore, understanding the mechanisms of hypertension that result from fetal programming will provide new interventional strategies for reducing the prevalence of hypertension. The current study has analyzed the susceptibility of hypertension in response to DOCA challenge in offspring of prenatal exposure to inflammation and the major findings are as follows: (1) prenatal exposure to LPS resulted in higher blood pressure and more severe vascular damage in adult offspring after postnatal 4 weeks of DOCA-salt treatment; (2) inadequate anti-oxidant defense capacity caused by impaired PGC-1α rapid recovery was implicated in vascular damage and blood pressure elevation in response to DOCA-salt treatment *via* oxidative stress in adult offspring of LPS-treated mothers; (3) mechanically, prenatal inflammation exposure compensatory increase in PGC-1α expression which in turn up-regulated the antioxidant capacity and maintained the O^2−^ below the subliminal level that could not cause obvious vascular damage in offspring’s thoracic aortas at a resting state. However, DOCA-salt treatment induced a sustained elevation of NF-κB activity and impaired the rapid PGC-1α recovery in thoracic aortas of offspring that received prenatal exposure to LPS, which may have a pivotal role in the early onset of hypertension.

Growing evidence has shown that exposure to detrimental factors during pregnancy sensitizes offspring to damage in adult^3^. For examples, viral infection during pregnancy increases the frequency of autism spectrum disorder in offspring^42^, maternal hyper-cholesterolaemia during pregnancy promotes offspring to develop atherosclerotic lesions at earlier age^43^ and high-fat diet feeding during pregnancy and lactation augmented offspring’s lung inflammation and remodeling^44^. Previous studies showed that 0.79 mg/kg LPS administration at gestational day 8, 10 and 12 caused a very low percentage of fetal anomalies with no obvious changes in abortion rates^45^,^46^. We previously found that this dose of LPS injection led to a slight blood pressure elevation but showed no significant effect on abortion, the size of pups and/or sex ratio of the litter^8^,^47^. The current study focuses on the effects of maternal inflammation on adult hypertension development. We found that higher blood pressure and more severe vascular damage in response to DOCA-salt stimulation in adult offspring of LPS-treated mothers, which adds more evidence for the concept of in utero origins of hypertension^48^. The traditional model of DOCA-salt induced hypertension in which the blood pressure increment is mainly mediated by increased intravascular volume and circulating catecholamines is dependent on unilateral nephrectomy^49^. Though renal function plays a critical role in maintaining normal blood pressure, the process of clinical hypertension development in respect to renal dysfunction could not be well imitated by unilateral nephrectomy in animal study^50^. The adult offspring of LPS-treated mothers in the current study showed increased inflammatory status and oxidative stress, although the blood pressure increment did not reach the high level to that obtained in DOCA-salt treatment plus nephrectomy. These offspring were prone to hypertension and vascular damage in the absence of nephrectomy when challenged with DOCA-salt. Epidemiological studies of primary hyper-aldosteronism being an important risk factor for hypertension^18^,^19^ encourage us to believe that DOCA-salt treatment without nephrectomy by using the adult offspring of LPS-treated mothers might be a good animal model for studying the mechanisms of hypertension development and also for pharmacodynamics evaluation of anti-hypertension drugs.

The progression of cardiovascular disease is usually accompanied by imbalance between generation and elimination of ROS, such as hypertension and atherosclerosis^51^. Previous studies showed that traditional DOCA-salt rats showed increased aortic O^2−^ production^52^ *via* increased expression of NADPH oxidase subunits p22phox, together with decreased anti-oxidant genes expression^24^. In the current study, despite the increased trend in the expression of NADPH oxidase subunits in control offspring after DOCA-salt challenge, the O^2−^ level was not significantly increased. This might be attributed to a healthy self-compensatory machinery of upregulated anti-oxidant defense capacity, as evidenced by the increased expression of UCP2 and Prdx5, though without any changes of SODs expression. However, there were not any induction of UCP2 and Prdx5 transcription, even with a significant reduction of SOD1 and SOD3 transcription, which is critical for the clearance of excessive ROS^53^, after DOCA-salt challenge in the thoracic aortas of offspring that received prenatal exposure to inflammation. The antioxidant NAC, which is an excellent source of sulfhydryl groups acting as a potential free radical scavenger^30^, could alleviate DOCA-salt induced blood pressure elevation and vascular damage in adult offspring of LPS-treated mothers. These results demonstrate that impaired motivation of anti-oxidant system existed in offspring of prenatal exposure to inflammation when suffering from secondary risk factors. A previous study showed that maternal high-fat diet repressed the expression of anti-oxidant defense genes in liver of the male offspring rats^26^. Furthermore, modification in antioxidant activity by maternal low protein diet could predispose to pancreatic islet dysfunction^54^. Our findings together with previous studied strongly suggested that the potential programming of ROS and anti-ROS system prenatally may be a vital risk factor for adult health in offspring of prenatal exposure to detrimental factors^26^,^54^.

PGC-1α is a crucial regulator in response to various stressors and associates with the development of several diseases^55^. Epidemiological studies have revealed that a common Gly482Ser polymorphism of PGC-1α is associated with arterial hypertension^56^. In addition, PGC-1α has also been reported to prevent the migration of vascular endothelial cells and smooth muscle cells and prevent endothelial dysfunction^57^,^58^. This may have an important role for PGC-1α in hypertension development. In our current study of control offspring, the protein level of PGC-1α was significantly increased after 4 weeks of DOCA treatment, which correlated with the increased expression of its direct target genes UCP2 and Prdx5^31^,^32^, supporting the critical role of PGC-1α in response to stressors. However, prenatal inflammatory stimulation led to a pre-existing higher level of PGC-1α in adult offspring at resting state, without any additional elevation after 4 weeks of DOCA treatment. The pre-existing higher level of PGC-1α might be an adaptive response to inflammation response^17^ during the prenatal stage and become programed in adult life, which in turn raises the threshold of anti-ROS response when suffering from a persistent weak ROS inducer.

Previous reports have revealed that a rapid PGC-1α rebound plays a protective role in renal functional recovery after sepsis^38^. Our current study also observed a similar trend in control offspring after DOCA-salt treatment. However, this rapid PGC-1α rebound did not exist in offspring of prenatal exposure to inflammation after DOCA-salt challenge. This demonstrates that the impaired rapid PGC-1α rebound, induced by prenatal exposure to inflammation or other detrimental factors, which was accompanied by increased ROS accumulation, renders the offspring more sensitive to vascular damage and the development of hypertension.

Mechanistically, we found that continued NF-κB over-activation contributed to the repressed PGC-1α rapid rebound in responding to DOCA-salt challenge in offspring of prenatal exposure to inflammation. This was consistent with previous studies that PGC-1α mRNA levels was markedly reduced after LPS injection and inflammation-dependent PGC-1α repression was rescued by inhibiting the NF-κB pathway^16^,^59^. This rapid rebound of PGC-1α expression in parallel with rapid NF-κB recovery at the early stage of DOCA-treatment in control offspring with DOCA-treatment suggests that they may antagonize each other, consistent with the slightly increased blood pressure and vascular damage. However, this mutual inhibition of them at the early stage of DOCA-salt challenge was substituted with persistent up-regulation of NF-κB activation during the whole period of DOCA-salt treatment in thoracic aortas of offspring that received prenatal exposure to inflammation, accompanied by retarded PGC-1α rebound. This NF-κB over-activation might attribute to the impaired NF-κB self-negative feedback loop on newly IκBα re-synthesis in the thoracic aortas of offspring that received prenatal inflammatory exposure^13^. This over-activation of NF-κB on inhibiting PGC-1α expression was further confirmed following the application of a NF-κB inhibitor, TPCK, which reversed the PGC-1α rebound, up-regulated the expression of anti-oxidant genes UCP2 and SOD1, as well as prevented blood pressure elevation after 7 days of DOCA-salt treatment. Therefore, proneness to NF-κB over-activation, programmed by prenatal inflammatory stimulation, plays a critical role in interrupting PGC-1α-mediated antioxidant capacity when responding to detrimental factors in adults.

Collectively, the present study has established a new re-stimulation model for studying the potential mechanisms of the early onset of hypertension by using offspring that received prenatal inflammatory exposure and postnatal DOCA-salt re-stimulation. Prenatal programmed proneness to persistent NF-κB over-activation impairs the role of PGC-1α on up-regulating antioxidant capacity, thereby having a critical role in the early onset of hypertension when responding to a second hypertensive risk factor. Since prenatal exposure to inflammation is an important unresolved public health problem worldwide and easily acquired experience of cardiovascular risk factors in adulthood, the present findings provide a new notion by which prenatal inflammatory stimulus contributes to the increased incidence of adult hypertension. New strategies based on restraining programmed NF-κB over-activation or antioxidant capacity will provide new targets for preventing the worldwide epidemic of hypertension.

## Methods and Materials

### Animals

Nulliparous time-dated pregnant SD rats were purchased from the Animal Centre of Third Military Medical University (Chongqing, China). All procedures were approved by the local animal ethics committee at Third Military Medical University in accordance with the principles outlined in the National Institutes of Health Guide for the Care and Use of Laboratory Animals. At the end of experiments, the rats were anaesthetized by 7% chloral hydrate (1 ml/200 g) and executed, and then thoracic aortas and superior mesenteric arteries were obtained.

### Prenatal inflammatory stimulation model

Time-dated pregnant SD rats (250 g to 300 g) were randomly divided into two groups as described previously^8^. The pregnant rats were intraperitoneally administered with saline (Control group) or LPS (0.79 mg/kg, Sigma-Aldrich, St. Louis, MO, USA) (LPS group), respectively, on gestational day (GD) 8, 10 and 12.

### Postnatal DOCA-salt stimulation model

Male pups from the aforementioned control and LPS group were subjected to 1% NaCl in the drinking water with subcutaneous injection of DOCA (20 mg/kg, Wako Pure Chemical Industries Ltd, Osaka, Japan) every other day from post-birth week 16 to week 20, defined as Con+DOCA and LPS+DOCA group, respectively. Control and LPS group treated with 50% glycerol subcutaneously were taken as vehicle control, defined as Con+Ve and LPS+Ve group, respectively. For NAC (Sigma-Aldrich) treatment, NAC (0.5 g/L, estimated oral intake of 187 mg/kg/d) in the drinking water was given to Con+DOCA and LPS+DOCA group for 4 weeks, defined as Con+DOCA+NAC and LPS+DOCA+NAC group, respectively. For TPCK (Sigma-Aldrich)^39^ treatment, TPCK (3 mg/kg,) was given daily to Con+DOCA or LPS+DOCA group intraperitoneally, defined as Con+DOCA+TPCK and LPS+DOCA+TPCK group, respectively.

### SBP measurement

SBP was measured by the noninvasive tail-cuff method with computer-assisted BP-2010 Series tail measurement equipment (Softron Beijing Biotechnology Co., Ltd, Beijing, China). The operation procedure was followed as described previously^8^. Briefly, rats were placed inside a warming chamber (~34 °C) for 15 minutes before measurement of artery blood pressure and then were placed in plastic restraints. A cuff with a pneumatic pulse sensor was attached to the tail. SBP was calculated from three consecutive recordings. The measurement of blood pressure was performed 9 to 11 am at the next day after DOCA treatment at indicated time points. 0 week means the day before the first time of DOCA treatment. The investigators were blinded for measuring the blood pressure.

### Histological analysis

Histological structures of thoracic aortas and superior mesenteric arteries were determined by standard HE staining. The coverslips were visualized under a Leica confocal laser-scanning microscope (Leica, Wetzlar, Germany). The values of vascular wall thickness, cross sectional area and wall:lumen ratio were quantified using NIS-Elements BR software (Nikon Corporation, Tokyo, Japan), as described elsewhere^60^. The investigators were blinded for acquiring the images.

### Immunofluorescence staining

The protein levels of p-eNOS^Ser1177^ and eNOS in thoracic aortas were determined by immunofluorescence staining as described previously^10^. Donkey Anti-Rabbit IgG H&L (Alexa Fluor^®^ 647) (Abcam, Cambridge, UK) was used for an additional 1 hour incubation after anti-p-eNOS^Ser1177^ or anti-eNOS (Abcam, Cambridge, UK) incubation and washing. Sections were then incubated with 1 mg/ml DAPI for 10 min, washed three times with PBS, and mounted into Vectashield^®^ mounting medium (Vector Laboratories, Burlingame, CA). The coverslips were visualized under a Leica confocal laser scanning microscope (Leica, Wetzlar, Germany). The investigators were blinded for acquiring the images. The value of fluorescence was quantified using Image J software (National Institutes of Health, Bethesda, MD, USA).

### Real-time RT-PCR

Real-time RT-PCR was performed as described previously^61^. Total RNA was extracted from thoracic aortas using Trizol (Roche Molecular Biochemicals, Mannheim, Germany) and total RNA (1 μg) was then reverse-transcribed into cDNA using a First Stand cDNA Synthesis Kit (DBI Bioscience, Ludwigshafen, Germany). Each Real-time PCR reaction was carried out in a total volume of 20 μl with Quanti Tect SYBR Green PCR Master Mix (MJ Research) according to the following conditions: 2 min at 95 °C, 40 cycles at 95 °C for 10 s, 60 °C for 15 s, 68 °C for 10 s, 72 °C for 20 s, using the ABI Prism 7700 sequence detection system (ABI). The cycle threshold (Ct) values were normalized by the internal control β-actin. Primer sequences for real-time RT-PCR, obtained from reported literatures or designed by Pubmed Primer-BLAST, were listed in Supplementary Table S1.

### *In Situ* O^2−^ detection

O^2−^ level was measured by DHE (Invitrogen, Carlsbad, CA, USA) staining, according to a previous report with some modification^62^. Briefly, cross segments of thoracic aortas were placed in a phosphate buffer solution buffer and allowed to equilibrate for 30 minutes at 37 °C, followed by incubated with 5 μmol/L DHE for additional 20 minutes at 37 °C in dark. After incubation, segments were washed with warmed phosphate buffer solution for 5 minutes X 3 times at 37 °C and then quickly imaged under a Leica DM4000B microscope (Leica). The value of DHE fluorescence was quantified using Image J software (National Institutes of Health, Bethesda, MD, USA).

### Immunoblotting

Immunoblotting was performed as described elsewhere^63^. Briefly, thoracic aortas were homogenized in a T-PER tissue protein extraction reagent (Thermo-Pierce, Rockford, IL, USA) with protease inhibitor cocktail (Sigma-Aldrich). Homogenates were centrifuged at 4 °C for 15 min at 12,000 g, and supernatants were collected. Protein was quantified using the protein assay kit from Bio-Rad. After diluted with 4 × Laemmli buffer (Bio-Rad) supplemented with 2.5% β-mercaptoethonal, boiled for 5 min, and samples were subjected to immunoblotting analysis. Samples with equal protein were loaded on 10% or 12% polyacrylamide gel supplementary with 0.1% sodium dodecyl sulfate (SDS) and separated by electrophoresis at 120 V. Proteins were then transferred onto PVDF membranes. Nonspecific binding sites in the membranes were blocked with 5% nonfat dry milk for 1 hour at room temperature. The membranes were then incubated with primary antibodies overnight at 4 °C, followed by a secondary antibody incubation. Proteins were visualized with enhanced chemi-luminescence reagents, and the blots were exposed to hyper film. Primary antibodies were used as followings: anti-phosphorylated (p)- -p65^Ser536^ (93H1), anti-p65 (D14E12), anti-p-p38 MAPK (Thr180/Tyr182), anti-p-ERK1/2 (Thr202/Tyr204), anti-p-JNK (Thr183/Tyr185) (Cell signaling Technology, Beverly, MA, USA); anti-PGC-1(H-300) (polyclonal), anti-UCP2 (N-19) (polyclonal) (Santa Cruz Biotechnology, Santa Cruz, CA, USA); anti-VCAM-1(324), anti-SOD3 (Abcam, Cambridge, UK); anti-ICAM-1(Polyclonal) (R&D Systems, Minneapolis, MN, USA), anti-SOD1 or anti-β-actin (AC-15) (Sigma-Aldrich). The single band for β-actin, under multiantigens, is a representative picture of several different blots with the same samples. Results were quantified by using Quantity-one software (Bio-Rad Laboratories, Hercules, CA, USA).

### Statistical analysis

Data are expressed as means ± S.E.M. Significance of difference in mean values was determined using a two-way ANOVA analysis of variance followed by the Bonferroni post hoc test. A *P* value less than 0.05 was considered to be significant.

## Additional Information

**How to cite this article**: Deng, Y. *et al*. Sustained elevation of NF-κB activity sensitizes offspring of maternal inflammation to hypertension *via* impairing PGC-1α recovery. *Sci. Rep.* **6**, 32642; doi: 10.1038/srep32642 (2016).

## Supplementary Material

Supplementary Information
