# Supplementary material for: Sustained elevation of NF-κB activity sensitizes offspring of maternal inflammation to hypertension via impairing PGC-1α recovery
Source: Sci Rep. 2016 Sep 12;6:32642. doi: 10.1038/srep32642 (PMC5018852; doi:10.1038/srep32642)
Supplement: Supplementary Information [file srep32642-s1.pdf]

Sustained elevation of NF-κB activity sensitizes offspring of maternal inflammation to hypertension *via* impairing PGC-1α recovery

Yafei Deng[1,2,8] , Qi Zhang[1,2,8] , Hongqin Luo[1,2,8], Xianhua Chen[3,8], Qi Han[1,2], Fangjie Wang[1,2], Pei Huang[1,2], Wenjing Lai[1,2], Xiao Guan[1,2], Xiaodong Pan[1,2], Yan Ji[1,2], Wei Guo[1,2], Ling Che[1,2],Yuan Tang[1,2], Liangqi Gu[4], Jianhua Yu[5], Michael Namaka[6,7], Youcai Deng[1,2,*], Xiaohui Li[1,2,*]

Supplementary Materials

Supplementary Figures and Figure Legend

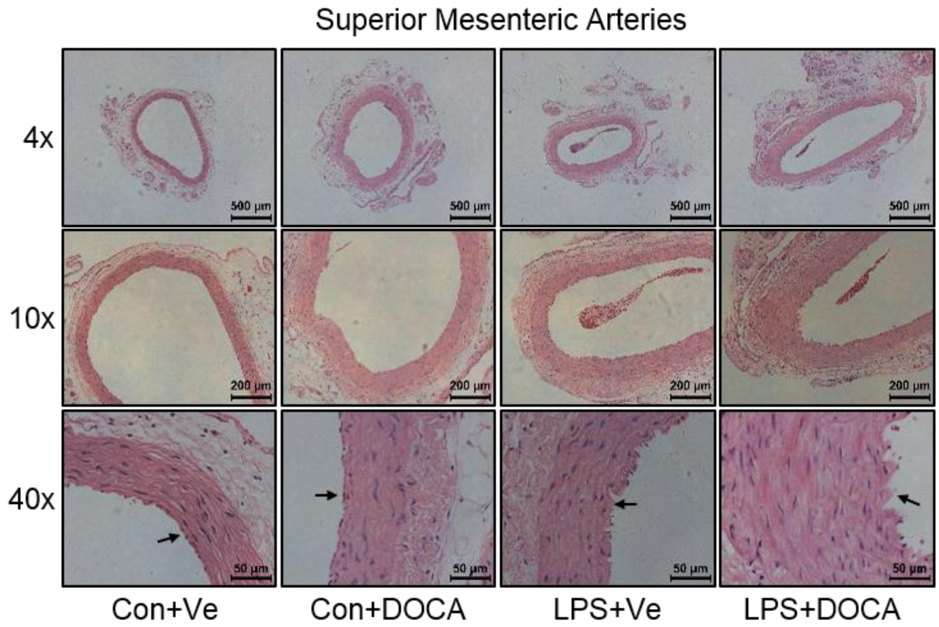

Supplemental Fig. S1. Effects of DOCA-salt treatment on superior mesenteric arteries in adult offspring of LPS-treated mothers. Offspring were treated as described in Fig. 1a and HE staining of superior mesenteric arteries were shown. The arrow direction represents endothelium and representative pictures from each group were shown. n=6 for each group.

(a)

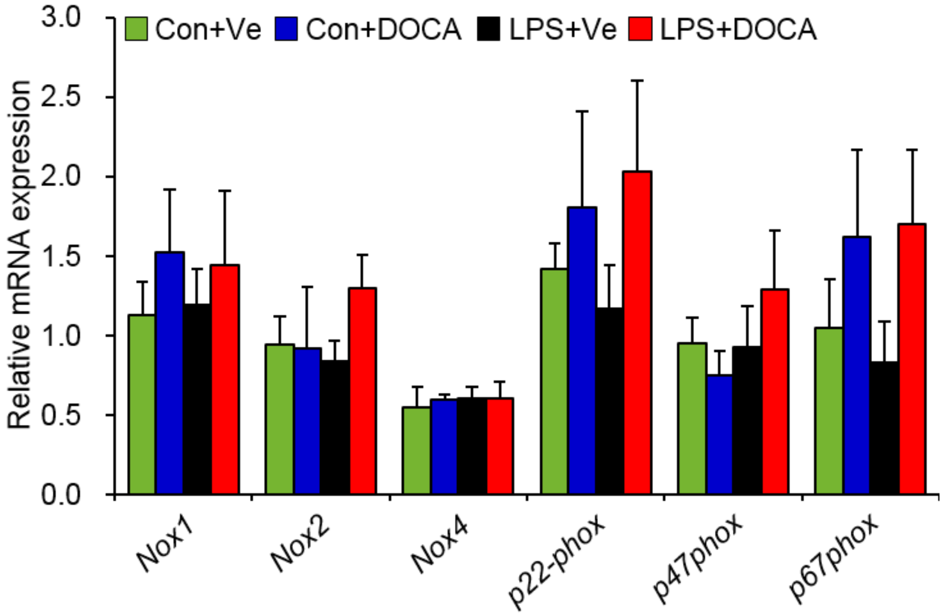

(b)

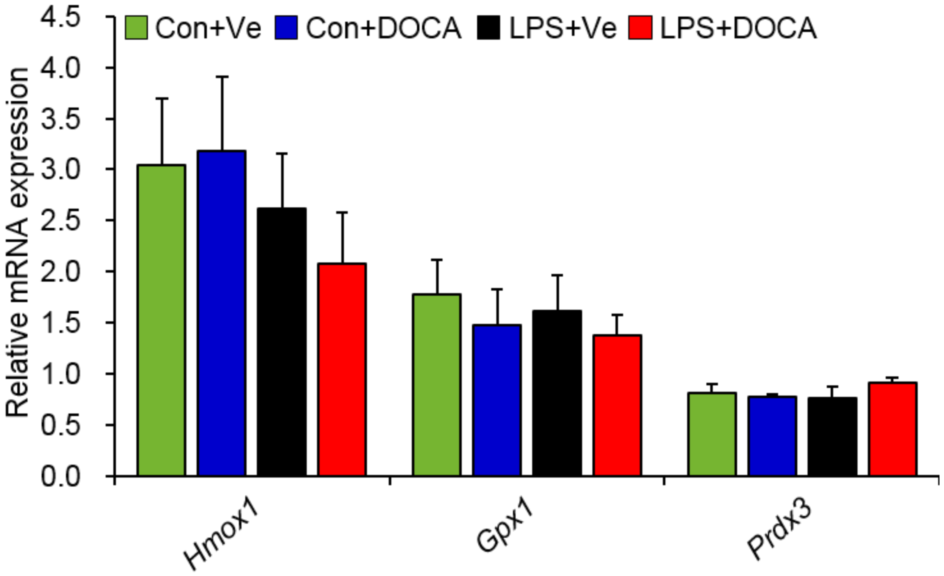

Supplemental Fig. S2. The mRNA expression of NADPH oxidase subunits and antioxidant–related genes in adult offspring of LPS-treated mothers after 4 weeks of DOCA-salt treatment. (**a**) and (**b**) Offspring were treated as described in Fig.1a and the mRNA levels of various subunits of NADPH oxidase (**a**) and antioxidant-related genes (**b**) in thoracic aortas were determined by real-time RT-PCR. *β-actin* was taken as internal control. Error bar represents S.E.M. n=6-7 for each group in (**a**) and (**b**).

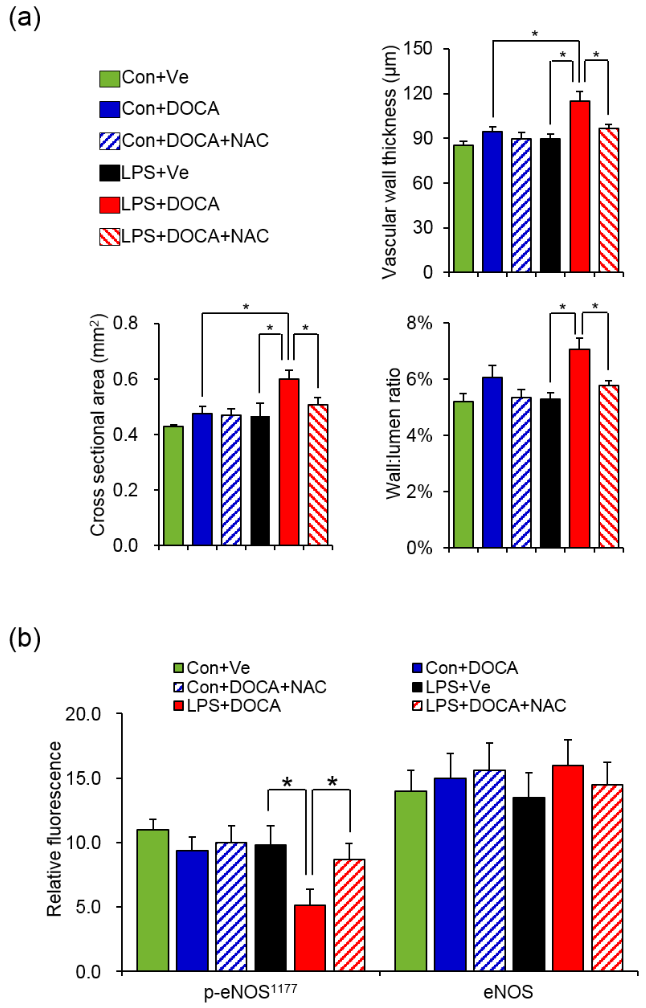

Supplemental Fig. S3 Antioxidant NAC prevents vascular damage in thoracic aortas of offspring that received prenatal exposure to LPS after 4 weeks of DOCA-salt treatment. (**a**) HE staining of thoracic aortas and representative pictures from each group were shown in Fig. 3b and the values of vascular wall thickness, cross sectional area and wall:lumen ratio were quantified using NIS-Elements BR software. (**b**) Immunofluorescence staining of the p-eNOS^{Ser1177} and eNOS protein levels and representative pictures from each group were shown in Fig.3c and the value of fluorescence was quantified using Image J software. Error bar represents S.E.M. **P*<0.05 denotes the statistical comparison between the two marked treatment groups. n=6 per group. Two-way ANOVA.

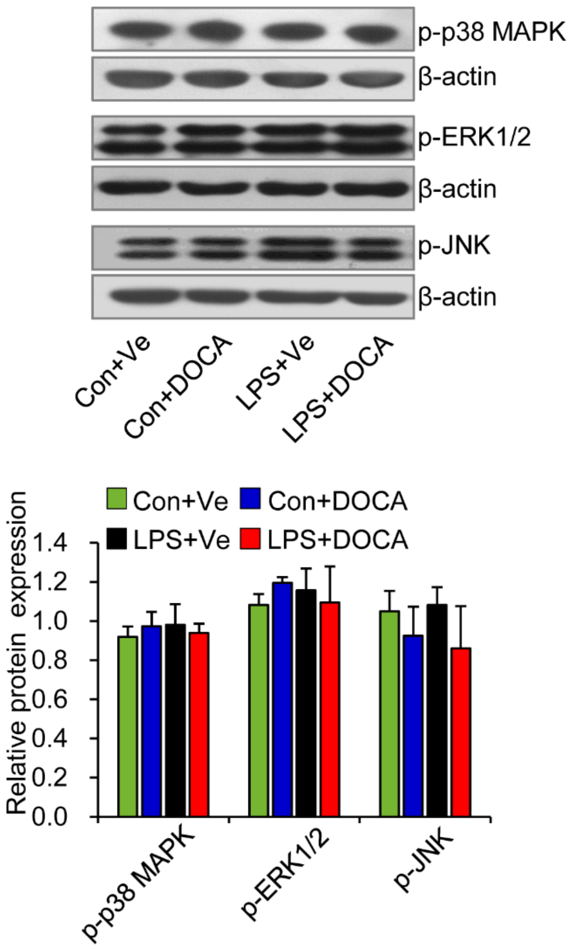

Supplemental Fig. S4. The MAPK signaling was not changed after 4 weeks of DOCA-salt treatment in adult offspring of LPS-treated mothers. Offspring were treated as described in Fig. 1a and the phosphorylation levels of anti-p-p38 MAPK, p-ERK1/2 and p-JNK in thoracic aortas were assessed by immunoblotting after 4 weeks of DOCA-salt treatment. Representative plots in each group and statistical data of relative densitometry, normalized by β-actin, were shown. n=6 per group.

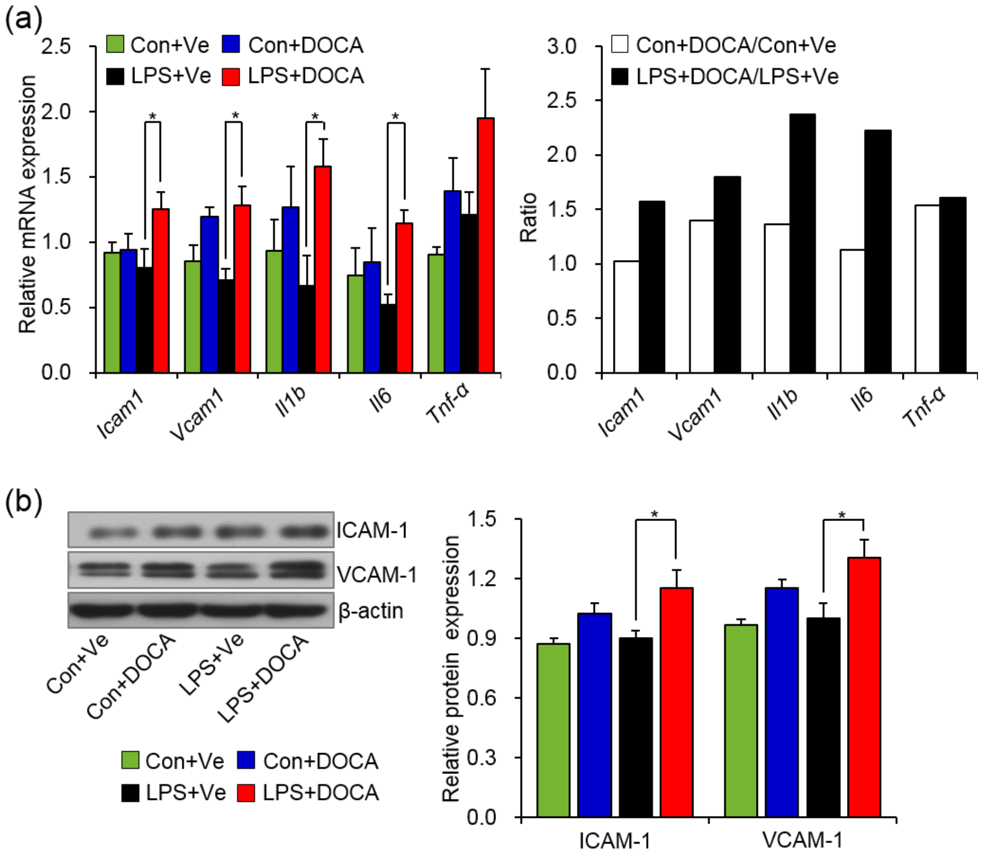

Supplemental Fig. S5. Prenatal exposure to LPS results in increased inflammatory factors in thoracic aortas of offspring after 4 weeks of DOCA-salt treatment. (**a**) Offspring were treated as described in Fig.1a and the mRNA levels of *Icam1*, *Vcam1*, *Il1b*, *Il6* and *Tnf-α* in thoracic aortas were determined by real-time RT-PCR. *β-actin* was taken as internal control. n = 6 - 7 per group. (**b**) Offspring were treated as described in Fig.1a and the protein expression levels of ICAM-1 and VCAM-1 in thoracic aortas were determined by immunoblotting in control and adult offspring of LPS-treated mothers after 4 weeks of DOCA-salt treatment. Representative plots in each group (left panel) and statistical data of relative densitometry, normalized by β-actin (right panel), were shown. n = 6 per group. Error bar represents S.E.M. *$P<0.05$ denotes the statistical comparison between the two marked treatment groups, respectively. Two-way ANOVA.

Table S1. Primers for real time RT-PCR

Gene symbol	Forward primer (5'-3')	Reverse primer (5'-3')
Sod1[1]	TGTGTCCATTGAAGATCGTGTGA	TCTTGTTTCTCGTGGACCACC
Sod2[1]	TTAACGCGCAGATCATGCA	CCTCGGTGACGTTCAGATTGT
Sod3	TTCCCAGACACCTCAATCGC	TCTGTGGAGTGCATAGGTGTG
Hmox1[2]	TCTATCGTGCTCGCATGAAC	CAGCTCCTCAAACAGCTCAA
Ucp2	GAGAGTCAAGG GCTAGCGC	GCTTCGACAGTGCTCTGGTA
p22-phox[3]	TGGCCTGATCCTCATCACAG	AGGCACGGACAGCAGTAAGT
p47phox[3]	TCACCGAGATCTACGAGTTC	TCCCATGAGGCTGTTGAAGT
p67phox (*Ncf2*)[1]	GCTTCGGAACATGGTGTCTAAGA	AGGGTCAGGCAGTAGTTTTTCACTTG
Nox1[4]	CTTCCTCACTGGCTGGGATA	CGACAGCATTTGCGCAGGCT
Nox2	GGAGTGGTGTGTGAATGC	TTTGGTGGAGGATGTGATGA
Nox4[5]	ACAGTCCTGGCTTACCTTCG	TTCTGGGATCCTCATTCTGG
Catalase (*Cat*)	AAACCCGATGTCCTGACCAC	CCTTTGCCTTGGAGTATCTGG
Gpx1	TCGAACCCGATATAGAAGCCC	CACCAAGCCCAGATACCAGG
Prdx3	GAAGGTTGCTCTGGTCCTCG	CAGCAGGGGTGTGGAATGAA
Prdx5	AGCTGAGGTTTTGCGTCCTA	GGTGTCTCCCACCTTGATCG
Icam1	CTTTGCCCTGGTCCTCCAAT	GTCTTCCCCAATGTCGCTCA

Vcam1	ACAAGGCTACATGAGGGTGC	AACGGAATCCCCAACCTGTG
Tnf-α (*Tnf*)	CAAGGCTGCCCCGACTATGTGC	TTGATGGCGGAGAGGAGGCTGAC
Il1b	AAGCTGTCTTCAGGCCAACA	CCCGTAGGGCGATTACAGTC
Il6	CTTCCAGCCAGTTGCCTTCTTG	GTCTGTTGTGGGTGGTATCCTC
β-actin (Actb)	GACGTTGACATCCGTAAAGACC	TAGGA GCCAGGGCAGTAATCT

References

1. Chabrashvili, T. *et al.* Effects of ANG II type 1 and 2 receptors on oxidative stress, renal NADPH oxidase, and SOD expression. *Am J Physiol Regul Integr Comp Physiol* **285**, R117-24 (2003).
2. Yoshida, T. *et al.* Monitoring changes in gene expression in renal ischemia-reperfusion in the rat. *Kidney Int* **61**, 1646-54 (2002).
3. Fan, C. *et al.* Synergy of aldosterone and high salt induces vascular smooth muscle hypertrophy through up-regulation of NOX1. *J Steroid Biochem Mol Biol* **111**, 29-36 (2008).
4. Uchizono, Y. *et al.* Expression of isoforms of NADPH oxidase components in rat pancreatic islets. *Life Sci* **80**, 133-9 (2006).
5. Zarzuelo, M.J. *et al.* SIRT1 inhibits NADPH oxidase activation and protects endothelial function in the rat aorta: implications for vascular aging. *Biochem Pharmacol* **85**, 1288-96 (2013).
